# Author Correction: Accelerated single cell seeding in relapsed multiple myeloma

**DOI:** 10.1038/s41467-021-20978-y

**Published:** 2021-01-20

**Authors:** Heather J. Landau, Venkata Yellapantula, Benjamin T. Diamond, Even H. Rustad, Kylee H. Maclachlan, Gunes Gundem, Juan Medina-Martinez, Juan Arango Ossa, Max F. Levine, Yangyu Zhou, Rajya Kappagantula, Priscilla Baez, Marc Attiyeh, Alvin Makohon-Moore, Lance Zhang, Eileen M. Boyle, Cody Ashby, Patrick Blaney, Minal Patel, Yanming Zhang, Ahmet Dogan, David J. Chung, Sergio Giralt, Oscar B. Lahoud, Jonathan U. Peled, Michael Scordo, Gunjan Shah, Hani Hassoun, Neha S. Korde, Alexander M. Lesokhin, Sydney Lu, Sham Mailankody, Urvi Shah, Eric Smith, Malin L. Hultcrantz, Gary A. Ulaner, Frits van Rhee, Gareth J. Morgan, Ola Landgren, Elli Papaemmanuil, Christine Iacobuzio-Donahue, Francesco Maura

**Affiliations:** 1 Adult Bone Marrow Transplant Service, Department of Medicine, Memorial Sloan Kettering Cancer Center, New York, NY USA; 2 Department of Medicine, Weill Cornell Medical College, New York, NY USA; 3 Department of Epidemiology and Biostatistics, Memorial Sloan Kettering Cancer Center, New York, NY USA; 4 Myeloma Service, Department of Medicine, Memorial Sloan Kettering Cancer Center, New York, NY USA; 5 Human Oncology & Pathogenesis Program, Memorial Sloan Kettering Cancer Center, New York, NY USA; 6 NYU Perlmutter Cancer Center, New York, NY USA; 7 Myeloma Center, University of Arkansas for Medical Sciences, Little Rock, AR USA; 8 Center for Hematological Malignancies, Department of Medicine, Memorial Sloan Kettering Cancer Center, New York, NY USA; 9 Cytogenetics Laboratory, Department of Pathology, Memorial Sloan Kettering Cancer Center, New York, NY USA; 10 Hematopathology Service, Department of Pathology, Memorial Sloan Kettering Cancer Center, New York, NY USA; 11 Department of Radiology, Memorial Sloan Kettering Cancer Center, New York, NY USA

**Keywords:** Cancer genomics, Chemotherapy, Myeloma

Correction to: *Nature Communications* 10.1038/s41467-020-17459-z, published online 17 July 2020.

The original version of this Article contained an error in the spelling of the author Marc Attiyeh, which was incorrectly given as Marc Attiye. This has now been corrected in both the PDF and HTML versions of the Article.

