# Supplementary material for: Accelerated single cell seeding in relapsed multiple myeloma
Source: Nat Commun. 2020 Jul 17;11:3617. doi: 10.1038/s41467-020-17459-z (PMC7368016; doi:10.1038/s41467-020-17459-z)
Supplement: Supplementary file 1 — Supplementary Information [file 41467_2020_17459_MOESM1_ESM.pdf]

SUPPLEMENTARY INFORMATION

Accelerated single cell seeding in relapsed multiple myeloma

Heather Landau et al.

Supplementary Figures

Supplementary Figure 1. Phylogenetic tree reconstruction of each newly diagnosed patient included in the WXS cohort. Early and late cluster numbers are reported with blue and yellow rectangle, respectively.

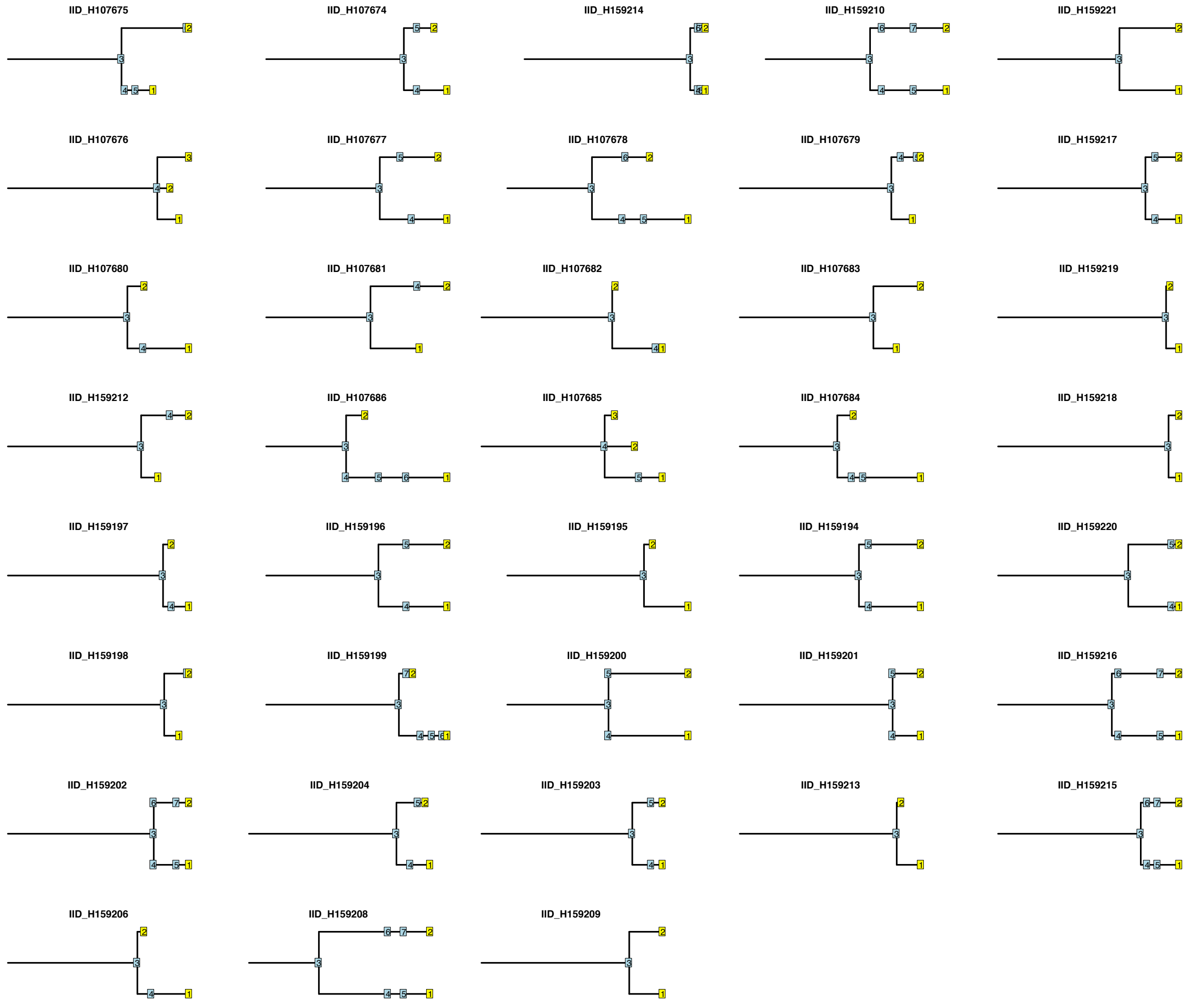

Supplementary Figure 2. Phylogenetic tree reconstruction of all patients with at least one sample collected at relapse included in the WXS cohort. Early and late cluster numbers are reported with blue and yellow rectangle, respectively.

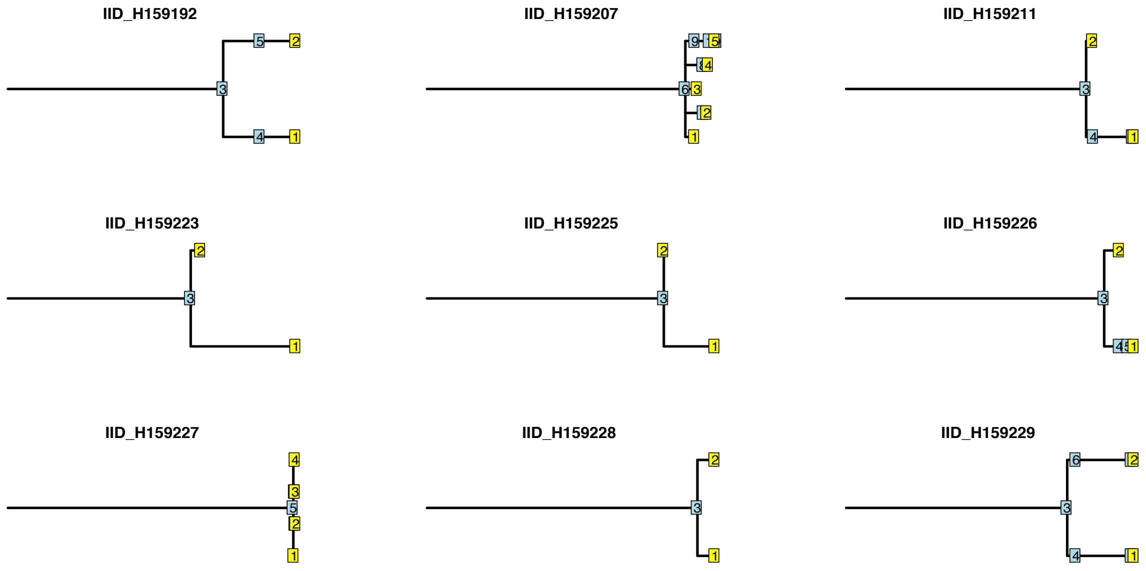

Supplementary Figure 3. Increased burden of nonsynonymous SNVs in relapsed multiple myeloma. a) Linear regression between the number of samples and evolutionary trajectories (number of clones); Blue, red and yellow dots represent relapsed WGS, relapsed WXS and newly diagnosed WXS samples, respectively. *lm* R package was used to estimate the p value ($p<0.0001$) and R square ($R^2=0.48$). b) Boxplot showing increased burden of nonsynonymous SNVs in relapsed multiple myeloma. p-values were estimated using Wilcoxon (*pairwise.wilcox.test* R function). WGS RR = whole genome sequencing at relapsed, WXS DG = whole exome sequencing at diagnosis, WXS RR = whole exome sequencing at relapsed. Boxplots show the median and interquartile range; observations outside this interval are shown as dots. c) Relative contribution to the R square in a multivariate linear regression model shown in (a). Estimates and 95%CI were generated using the *relaimpo* R package.

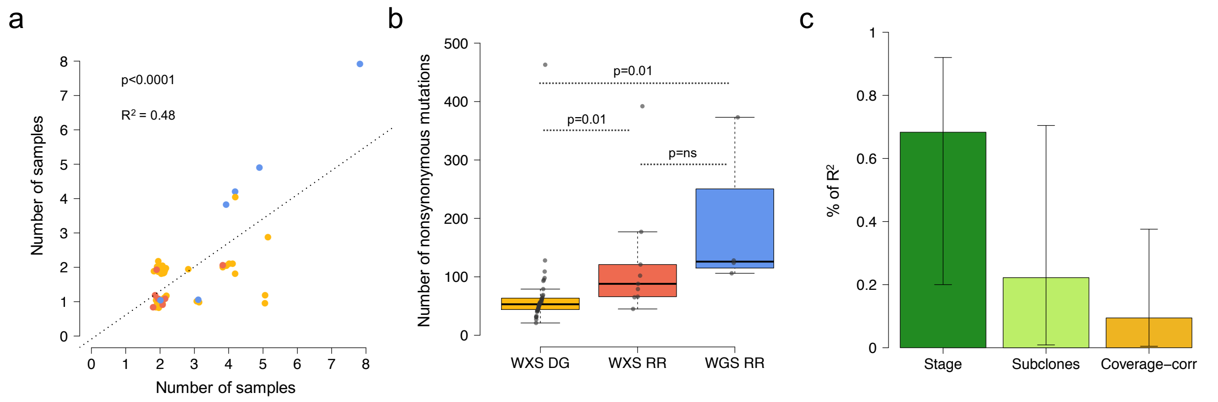

Supplementary Figure 4. The 8 mutational signatures extracted by SigProfiler. On the top left of each 96-mutational profile is annotated the signature(s) that contribute to the profile.

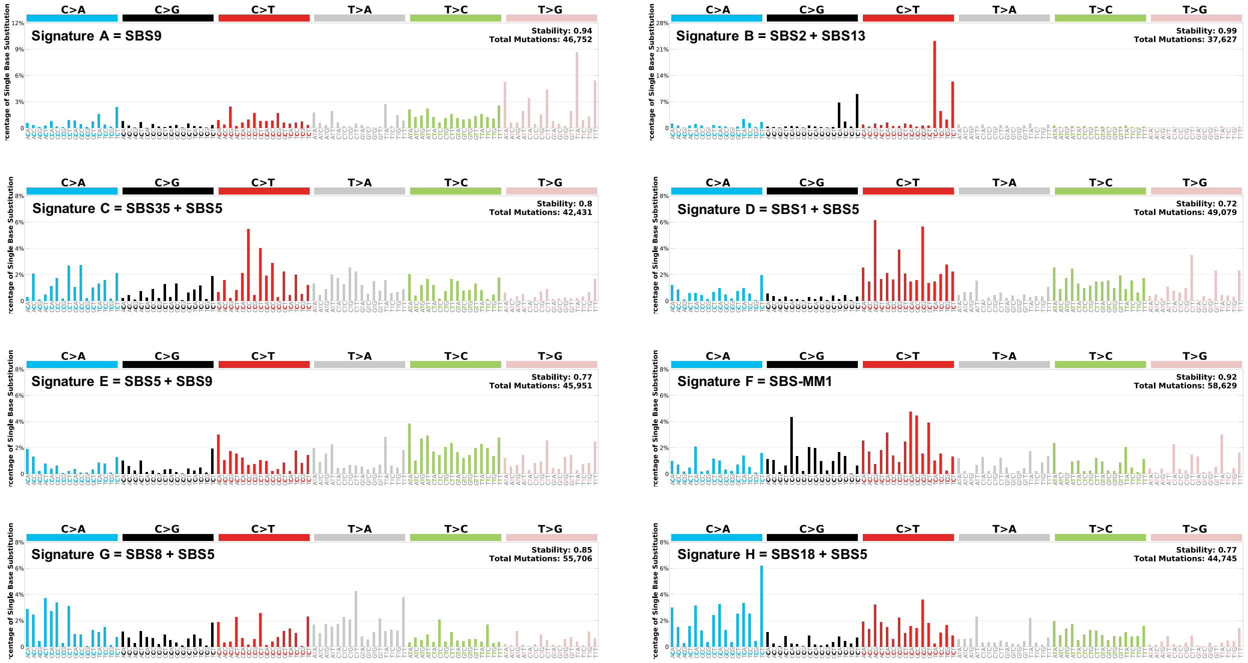

Supplementary Figure 5. The 96-mutational profile and mutational contribution of the trunks and the branches of all newly diagnosed and relapsed multiple myeloma included in the WXS cohort. The asterisk in d reflect the presence of transcriptional strand bias for SBS-MM1. Confidence interval of each mutational signature was generated by drawing 1000 mutational profiles from the multinomial distribution, each time repeating the signature fitting procedure, and finally taking the 2.5th and 97.5th percentile for each signature.

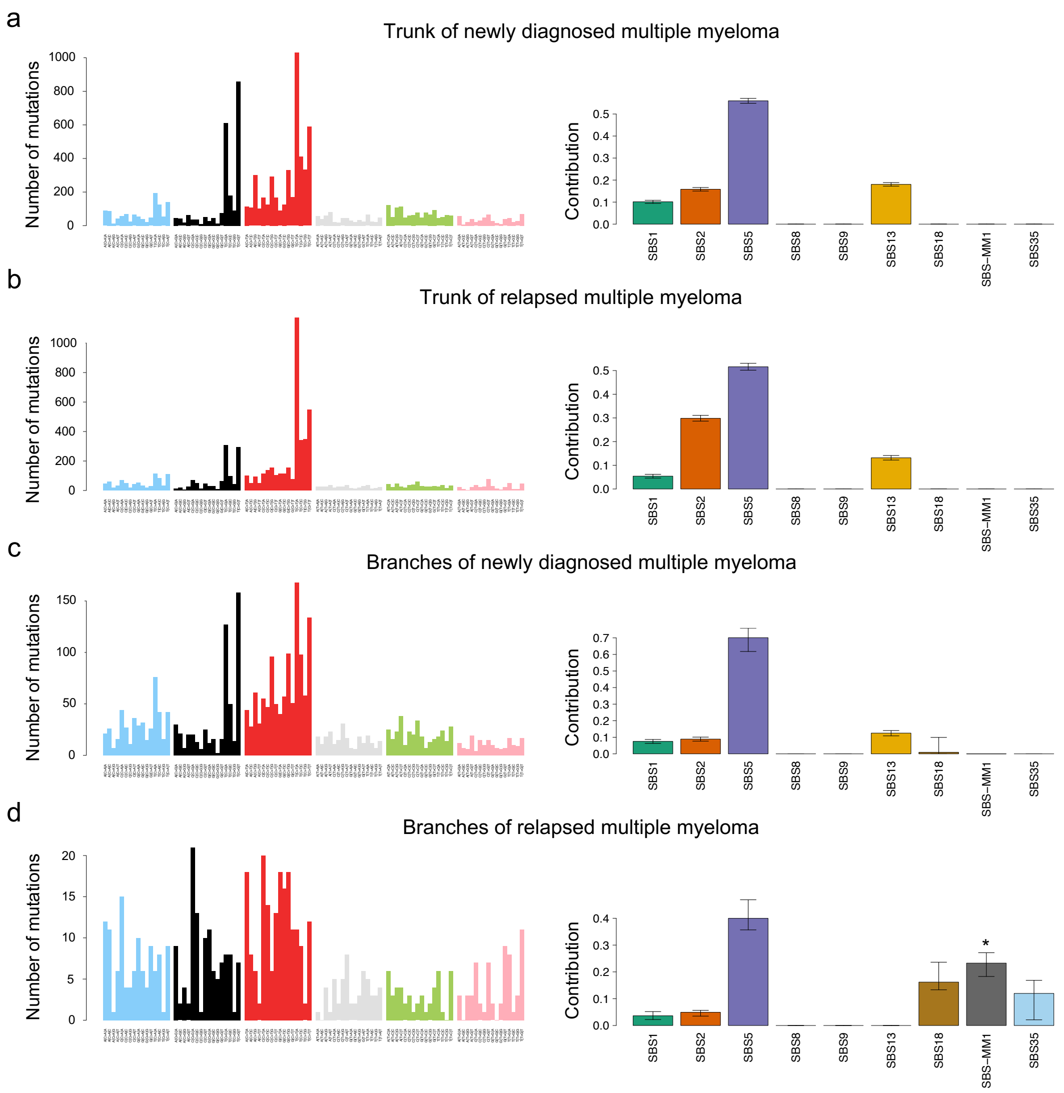

Supplementary Figure 6. Difference in SBS1 branches:trunk ratio between WXS at diagnosis, whole genome (WGS) and exome (WXS) relapsed multiple myeloma. p-value were estimated using Wilcoxon test (p=0.01). DG= diagnosis; RR= relapse. Boxplots show the median and interquartile range; observations outside this interval are shown as dots.

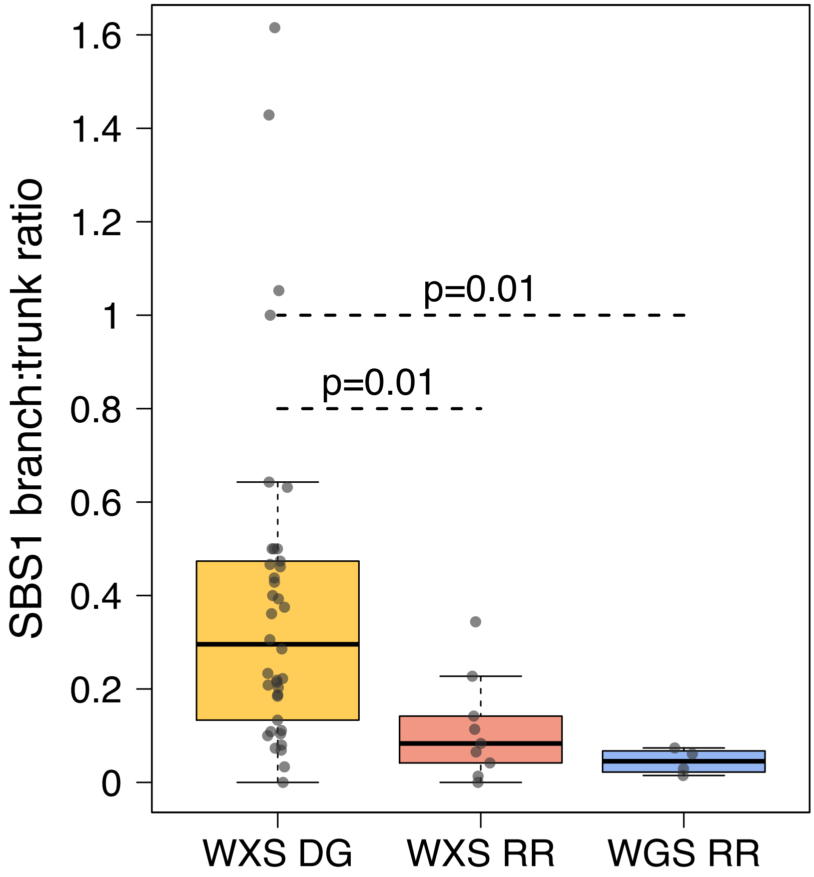

Supplementary Tables

Supplementary Table 1. WGS cohort demographic data.

Patients	Gender	Isotype	Age
**I-H-106917**	Male	IgG Kappa	53
**I-H-130718**	Male	IgG Kappa	60
**I-H-130719**	Male	IgG Lambda	54
**I-H-130720**	Male	IgA Kappa	64

Supplementary Table 2. Coverage, purity and ploidy of the WGS cohort.

Sample	Purity	Ploidy	Median Coverage
**I-H-106917-T2-1-D1-2**	0.6678	2.53	88.3745
**I-H-106917-T2-2-D1-2**	0.76726185	2.89423837	129.051
**I-H-106917-T2-3-D1-2**	0.78763	2.6048	101.449
**I-H-106917-T2-4-D1-2**	0.77841	2.4564	145.224
**I-H-130718-T1-1-D1-2**	0.94771604	2.15905083	89.9814
**I-H-130718-T1-10-D1-2**	0.9108	2.15	88.266
**I-H-130718-T1-11-D1-1**	0.74708	2.1824	121.605
**I-H-130718-T1-12-D1-1**	0.7	2.25	132.268
**I-H-130718-T1-2-D1-2**	0.92814	2.15	98.4156
**I-H-130718-T1-4-D1-2**	0.92	2.15	87.7814
**I-H-130718-T1-6-D1-2**	0.96419548	2.24056118	89.4443
**I-H-130718-T1-9-D1-2**	0.82908	2.2	89.8941
**I-H-130719-T1-2-D1-2**	0.8159	2.35	109.137
**I-H-130719-T1-4-D1-2**	0.2755	2.4	92.357
**I-H-130719-T1-5-D1-2**	0.69156	2.35	89.085
**I-H-130719-T1-6-D1-2**	0.68136	2.4	99.8962
**I-H-130720-T1-2-D1-2**	0.97924447	2.04353711	91.9502
**I-H-130720-T1-3-D1-2**	0.9598966	2.01730807	93.0744
**I-H-130720-T1-4-D1-2**	0.92907	2.05	88.1103
**I-H-130720-T1-5-D1-2**	0.97575174	2.05223504	88.2675
**I-H-130720-T1-8-D1-2**	0.95006146	2.02236114	88.3816
**I-H-130720-T1-9-D1-1**	0.26769043	2.8866776	107.828

Supplementary Table 3. Clinical and spatial characteristic of the whole exome cohort.

Study ID	Sample	Stage	Platinum-based	Melphalan-based	Posterior iliac crest	Biopsy
**1**	IID_H107686	Diagnosis	-	-	left	L4
**2**	IID_H107681	Diagnosis	-	-	left	Ilium
**3**	IID_H107677	Diagnosis	-	-	right	Symphysis
**4**	IID_H107675	Diagnosis	-	-	right	Ilium
**5**	IID_H107685	Diagnosis	-	-	right	Ilium
**6**	IID_H107683	Diagnosis	-	-	left	Sacrum
**7**	IID_H107679	Diagnosis	-	-	right	T8, Ilium
**8**	IID_H107682	Diagnosis	-	-	left	Rib, Pelvis, L1
**9**	IID_H107678	Diagnosis	-	-	left	Ilium
**10**	IID_H107684	Diagnosis	-	-	right	L1
**11**	IID_H107674	Diagnosis	-	-	left	L4
**12**	IID_H107676	Diagnosis	-	-	left	T5, Sacrum, Ilium x 2
**13**	IID_H107680	Diagnosis	-	-	right	T8
**14**	IID_H159193	Diagnosis	-	-	right	Iliac
**15**	IID_H159194	Diagnosis	-	-	right	Sacrum, Sacrum
**16**	IID_H159195	Diagnosis	-	-	right	T5
**17**	IID_H159196	Diagnosis	-	-	left	Acetabulum
**18**	IID_H159197	Diagnosis	-	-	left	Ilium
**19**	IID_H159198	Diagnosis	-	-	right	Clavicle
**20**	IID_H159199	Diagnosis	-	-	right	T12, T8, Acetabulum
**21**	IID_H159200	Diagnosis	-	-	left	L3
**22**	IID_H159201	Diagnosis	-	-	left	L5
**23**	IID_H159202	Diagnosis	-	-	left	Sacrum
**24**	IID_H159203	Diagnosis	-	-	right	T7
**25**	IID_H159204	Diagnosis	-	-	left	T8, L2, L3
**26**	IID_H159205	Diagnosis	-	-	left	Sacrum
**27**	IID_H159206	Diagnosis	-	-	left	L1
**28**	IID_H159207	Diagnosis	-	-	left	Ischium, Ilium
**28**	IID_H159207	Diagnosis	no	no	right	Pelvis
**29**	IID_H159208	Diagnosis	-	-	left	Sacrum
**30**	IID_H159209	Diagnosis	-	-	left	Anterior
**31**	IID_H159210	Diagnosis	-	-	left	Ilium, T10, L1
**48**	IID_H159227	Diagnosis	no	no	left	Ischium, Ilium, T8
**32**	IID_H159211	Diagnosis	-	-	right	Sacrum
**33**	IID_H159212	Diagnosis	-	-	right	Sacrum
**34**	IID_H159213	Diagnosis	-	-	right	Pleural
**35**	IID_H159214	Diagnosis	-	-	left	Posterior
**38**	IID_H159217	Diagnosis	-	-	right	Sacrum
**39**	IID_H159218	Diagnosis	-	-	right	Sacrum
**40**	IID_H159219	Diagnosis	-	-	right	Ilium
**41**	IID_H159220	Diagnosis	-	-	left	T8
**42**	IID_H159221	Diagnosis	-	-	left	Posterior
**45**	IID_H159224	Relapse	yes	no	-	T3 x2
**32**	IID_H159211	Relapse	yes	yes	right	Sacrum
**43**	IID_H159222	Relapse	yes	yes	right	Sacrum, T9
**44**	IID_H159223	Relapse	yes	yes	right	Sacrum

**46**	IID_H159225	Relapse	yes	yes	left	L4
**47**	IID_H159226	Relapse	yes	yes	left	Sacrum
**49**	IID_H159228	Relapse	yes	yes	left	Right
**50**	IID_H159229	Relapse	yes	yes	right	L2, Ilium
**51**	IID_H159192	Relapse	yes	yes	right	T12

Supplementary Table 4. Coverage and purity characteristics of the whole exome cohort.

Sample	Patients	Study ID	Purity	Ploidy	Median Coverage
**IID_H107674_T05_01_WE01**	IID_H107674	11	0.8002272	2.2694711	128
**IID_H107674_T06_01_WE01**	IID_H107674	11	0.6091187	2.2481825	116
**IID_H107675_T05_01_WE01**	IID_H107675	4	0.60931558	1.86780442	159
**IID_H107675_T06_01_WE01**	IID_H107675	4	0.50668536	1.93331034	99
**IID_H107676_T05_01_WE01**	IID_H107676	12	0.97001951	2.09682618	88
**IID_H107676_T06_01_WE01**	IID_H107676	12	0.96428541	2.09482283	82
**IID_H107676_T07_01_WE01**	IID_H107676	12	0.94218106	2.13657702	70
**IID_H107676_T08_01_WE01**	IID_H107676	12	0.95948579	2.12620523	87
**IID_H107676_T09_01_WE01**	IID_H107676	12	0.50818037	2.20878185	82
**IID_H107677_T05_01_WE01**	IID_H107677	3	0.8665444	2.05411238	73
**IID_H107677_T06_01_WE01**	IID_H107677	3	0.74822104	2.01652858	124
**IID_H107678_T05_01_WE01**	IID_H107678	9	0.94894169	1.97271947	80
**IID_H107678_T06_01_WE01**	IID_H107678	9	0.89273631	1.97978789	82
**IID_H107679_T05_01_WE01**	IID_H107679	7	0.5838939	2.3652043	59
**IID_H107679_T06_01_WE01**	IID_H107679	7	0.8354561	2.3706632	62
**IID_H107679_T07_01_WE01**	IID_H107679	7	0.8047266	2.3729548	62
**IID_H107680_T05_01_WE01**	IID_H107680	13	0.8438806	2.1559941	86
**IID_H107680_T06_01_WE01**	IID_H107680	13	0.8333836	2.1744959	91
**IID_H107681_T05_01_WE01**	IID_H107681	2	0.9630114	2.3173836	98
**IID_H107681_T06_01_WE01**	IID_H107681	2	0.6284588	2.5577561	103
**IID_H107682_T05_01_WE01**	IID_H107682	8	0.8939267	2.177801	82
**IID_H107682_T06_01_WE01**	IID_H107682	8	0.921053	2.1734415	83
**IID_H107682_T07_01_WE01**	IID_H107682	8	0.8197613	2.176688	125
**IID_H107682_T08_01_WE01**	IID_H107682	8	0.9217819	2.2564858	127
**IID_H107683_T04_01_WE01**	IID_H107683	6	0.7353756	2.5173613	110
**IID_H107683_T05_01_WE01**	IID_H107683	6	0.7644068	2.5169572	136
**IID_H107684_T05_01_WE01**	IID_H107684	10	0.73348935	1.9112504	90
**IID_H107684_T06_01_WE01**	IID_H107684	10	0.94217719	1.99039462	65
**IID_H107685_T05_01_WE01**	IID_H107685	5	0.8603543	2.6715024	111
**IID_H107685_T06_01_WE01**	IID_H107685	5	0.560074	2.3485615	73
**IID_H107686_T05_01_WE01**	IID_H107686	1	0.94766558	1.93627571	110
**IID_H107686_T06_01_WE01**	IID_H107686	1	0.6659103	2.2234326	100
**IID_H159192_T01_01_WE01**	IID_H159192	51	0.83804261	2.07896957	95
**IID_H159192_T02_01_WE01**	IID_H159192	51	0.82906367	2.08565467	95
**IID_H159193_T01_01_WE01**	IID_H159193	14	0.2853044	1.6461001	77
**IID_H159193_T02_01_WE01**	IID_H159193	14	0.2793778	1.70384482	77
**IID_H159194_T01_01_WE01**	IID_H159194	15	0.949149	2.1787565	71
**IID_H159194_T02_01_WE01**	IID_H159194	15	0.97119946	2.13396304	91

**IID_H159194_T03_01_WE01**	IID_H159194	15	0.90459305	2.14247739	79
**IID_H159195_T01_01_WE01**	IID_H159195	16	0.95128944	2.01012549	66
**IID_H159195_T02_01_WE01**	IID_H159195	16	0.93960744	1.99212335	85
**IID_H159196_T01_01_WE01**	IID_H159196	17	0.8384426	2.3350139	105
**IID_H159196_T02_01_WE01**	IID_H159196	17	0.4478289	2.29280087	84
**IID_H159197_T01_01_WE01**	IID_H159197	18	0.9224657	2.5700855	98
**IID_H159197_T02_01_WE01**	IID_H159197	18	0.8507106	2.539911	114
**IID_H159198_T01_01_WE01**	IID_H159198	19	0.94679	2.3540743	90
**IID_H159198_T02_01_WE01**	IID_H159198	19	0.8608714	2.3666977	90
**IID_H159199_T01_01_WE01**	IID_H159199	20	0.7013097	2.400672	96
**IID_H159199_T02_01_WE01**	IID_H159199	20	0.8488204	2.3950974	97
**IID_H159199_T03_01_WE01**	IID_H159199	20	0.9053691	2.3947028	82
**IID_H159199_T04_01_WE01**	IID_H159199	20	0.8603973	2.3947498	94
**IID_H159200_T01_01_WE01**	IID_H159200	21	0.96572423	2.04134535	133
**IID_H159200_T02_01_WE01**	IID_H159200	21	0.86389517	2.04768843	131
**IID_H159201_T01_01_WE01**	IID_H159201	22	0.49243245	1.99047269	143
**IID_H159201_T02_01_WE01**	IID_H159201	22	0.76782059	1.97094179	153
**IID_H159202_T01_01_WE01**	IID_H159202	23	0.8718508	2.206232	89
**IID_H159202_T02_01_WE01**	IID_H159202	23	0.7162207	2.2211288	123
**IID_H159203_T01_01_WE01**	IID_H159203	24	0.7806844	2.4000219	91
**IID_H159203_T02_01_WE01**	IID_H159203	24	0.8748083	2.4049286	95
**IID_H159204_T01_01_WE01**	IID_H159204	25	0.662403	2.230165	105
**IID_H159204_T02_01_WE01**	IID_H159204	25	0.68983017	2.19421531	133
**IID_H159204_T03_01_WE01**	IID_H159204	25	0.7695526	2.196245	126
**IID_H159204_T04_01_WE01**	IID_H159204	25	0.6537956	2.223384	117
**IID_H159205_T01_01_WE01**	IID_H159205	26	0.1108276	2.6798439	75
**IID_H159205_T02_01_WE01**	IID_H159205	26	0.95525302	2.06748533	78
**IID_H159206_T01_01_WE01**	IID_H159206	27	0.6056648	2.3623408	65
**IID_H159206_T02_01_WE01**	IID_H159206	27	0.7959656	2.3905351	77
**IID_H159207_T01_01_WE01**	IID_H159207	28	0.7339352	4.2380534	114
**IID_H159207_T01_01_WE01**	IID_H159207	28	0.7339352	4.2380534	114
**IID_H159207_T02_01_WE01**	IID_H159207	28	0.8440228	4.1297769	119
**IID_H159207_T02_01_WE01**	IID_H159207	28	0.8440228	4.1297769	119
**IID_H159207_T03_01_WE01**	IID_H159207	28	0.7653815	2.4118943	141
**IID_H159207_T03_01_WE01**	IID_H159207	28	0.7653815	2.4118943	141
**IID_H159207_T04_01_WE01**	IID_H159207	28	0.5479146	4.457577	103
**IID_H159207_T04_01_WE01**	IID_H159207	28	0.5479146	4.457577	103
**IID_H159207_T05_01_WE01**	IID_H159207	28	0.4643885	2.5469944	118
**IID_H159207_T05_01_WE01**	IID_H159207	28	0.4643885	2.5469944	118
**IID_H159208_T01_01_WE01**	IID_H159208	29	0.7877964	2.00249734	80
**IID_H159208_T02_01_WE01**	IID_H159208	29	0.79442943	1.9885857	84
**IID_H159209_T01_01_WE01**	IID_H159209	30	0.9637269	2.1989709	114

**IID_H159209_T02_01_WE01**	IID_H159209	30	0.7879392	2.1929875	126
**IID_H159210_T01_01_WE01**	IID_H159210	31	0.92712086	2.00858273	83
**IID_H159210_T02_01_WE01**	IID_H159210	31	0.93806964	1.97343159	107
**IID_H159210_T03_01_WE01**	IID_H159210	31	0.91711508	1.99323632	98
**IID_H159210_T04_01_WE01**	IID_H159210	31	0.86551271	1.96390226	120
**IID_H159211_T01_01_WE01**	IID_H159211	32	0.8783528	2.03188819	137
**IID_H159211_T01_01_WE01**	IID_H159211	32	0.8783528	2.03188819	137
**IID_H159211_T02_01_WE01**	IID_H159211	32	0.63023737	2.04069739	131
**IID_H159211_T02_01_WE01**	IID_H159211	32	0.63023737	2.04069739	131
**IID_H159211_T03_01_WE01**	IID_H159211	32	0.56188275	2.01511523	176
**IID_H159211_T03_01_WE01**	IID_H159211	32	0.56188275	2.01511523	176
**IID_H159211_T04_01_WE01**	IID_H159211	32	0.81070812	2.01084752	104
**IID_H159211_T04_01_WE01**	IID_H159211	32	0.81070812	2.01084752	104
**IID_H159212_T01_01_WE01**	IID_H159212	33	0.8652078	2.2682235	114
**IID_H159212_T02_01_WE01**	IID_H159212	33	0.5051255	2.27083046	115
**IID_H159213_T01_01_WE01**	IID_H159213	34	0.96980921	1.96990208	102
**IID_H159213_T02_01_WE01**	IID_H159213	34	0.75708204	1.97584576	125
**IID_H159214_T01_01_WE01**	IID_H159214	35	0.7750537	2.4587212	75
**IID_H159214_T02_01_WE01**	IID_H159214	35	0.7905361	2.461937	73
**IID_H159214_T03_01_WE01**	IID_H159214	35	0.7797573	2.4679043	70
**IID_H159215_T01_01_WE01**	IID_H159215	36	0.92918503	2.01415484	98
**IID_H159215_T02_01_WE01**	IID_H159215	36	0.69855941	2.01105364	99
**IID_H159216_T01_01_WE01**	IID_H159216	37	0.8330706	2.1822886	129
**IID_H159216_T02_01_WE01**	IID_H159216	37	0.82611333	2.15428486	135
**IID_H159217_T01_01_WE01**	IID_H159217	38	0.716789	1.9613428	133
**IID_H159217_T02_01_WE01**	IID_H159217	38	0.6839017	2.0669228	107
**IID_H159218_T01_01_WE01**	IID_H159218	39	0.94526328	1.93725482	88
**IID_H159218_T02_01_WE01**	IID_H159218	39	0.93183277	1.93351012	84
**IID_H159219_T01_01_WE01**	IID_H159219	40	0.89509	1.8119822	77
**IID_H159219_T02_01_WE01**	IID_H159219	40	0.572952	1.82642918	113
**IID_H159220_T01_01_WE01**	IID_H159220	41	0.8937164	2.2900012	99
**IID_H159220_T02_01_WE01**	IID_H159220	41	0.8173879	2.2983591	95
**IID_H159221_T01_01_WE01**	IID_H159221	42	0.8370796	2.6851918	117
**IID_H159221_T02_01_WE01**	IID_H159221	42	0.9003912	2.7185433	110
**IID_H159222_T01_01_WE01**	IID_H159222	43	0.743228	3.047736	77
**IID_H159222_T02_01_WE01**	IID_H159222	43	0.7252859	3.1409718	79
**IID_H159222_T03_01_WE01**	IID_H159222	43	0.79253508	1.86817258	84
**IID_H159223_T01_01_WE01**	IID_H159223	44	0.96868429	2.11916229	95
**IID_H159223_T02_01_WE01**	IID_H159223	44	0.47157677	2.22985945	77
**IID_H159224_T01_01_WE01**	IID_H159224	45	0.8738939	2.609495	66
**IID_H159224_T02_01_WE01**	IID_H159224	45	0.7960685	4.6081039	95
**IID_H159225_T01_01_WE01**	IID_H159225	46	0.5272777	1.9398966	102

**IID_H159225_T02_01_WE01**	IID_H159225	46	0.47170094	1.93603211	120
**IID_H159226_T01_01_WE01**	IID_H159226	47	0.92537319	1.99920882	96
**IID_H159226_T02_01_WE01**	IID_H159226	47	0.53255266	1.91373547	123
**IID_H159227_T01_01_WE01**	IID_H159227	48	0.9107675	2.3705624	117
**IID_H159227_T02_01_WE01**	IID_H159227	48	0.9751333	2.3660556	115
**IID_H159227_T03_01_WE01**	IID_H159227	48	0.9383707	2.3171541	129
**IID_H159227_T04_01_WE01**	IID_H159227	48	0.7306523	2.3878758	115
**IID_H159228_T01_01_WE01**	IID_H159228	49	0.9660542	2.3084885	103
**IID_H159228_T02_01_WE01**	IID_H159228	49	0.8801174	2.2496426	96
**IID_H159229_T01_01_WE01**	IID_H159229	50	0.8335068	2.2233683	67
**IID_H159229_T02_01_WE01**	IID_H159229	50	0.9234834	2.2469325	66
**IID_H159229_T03_01_WE01**	IID_H159229	50	0.8911536	2.2505918	67

Supplementary Table 5. *SigProfiler* assignment of mutational signatures.

De novo extracted	Global NMF Signatures
**Signature 96-A**	Signature SBS9 (94.66%)
**Signature 96-B**	Signature SBS2 (64.42%) & Signature SBS13 (35.58%)
**Signature 96-C**	Signature SBS5 (57.54%) & Signature SBS35 (42.46%)
**Signature 96-D**	Signature SBS1 (17.80%) & Signature SBS5 (75.48%)
**Signature 96-E**	Signature SBS5 (63.62%) & Signature SBS9 (25.56%)
**Signature 96-F**	Signature SBS-MM1
**Signature 96-G**	Signature SBS5 (27.52%) & Signature SBS8 (72.48%)
**Signature 96-H**	Signature SBS5 (47.20%) & Signature SBS18 (40.36%)
