# Supplementary material for: Accelerated single cell seeding in relapsed multiple myeloma
Source: Nat Commun. 2020 Jul 17;11:3617. doi: 10.1038/s41467-020-17459-z (PMC7368016; doi:10.1038/s41467-020-17459-z)
Supplement: Supplementary file 3 — Description of Additional Supplementary Files [file 41467_2020_17459_MOESM3_ESM.pdf]

**Description of Additional Supplementary Files**

File Name: Supplementary Data 1
Description: Summary of the key drivers in each sample included in the study

File Name: Supplementary Data 2
Description: Biopsy sites and PET-CT correlates for each patient
