# Supplementary material for: Accelerated single cell seeding in relapsed multiple myeloma
Source: Nat Commun. 2020 Jul 17;11:3617. doi: 10.1038/s41467-020-17459-z (PMC7368016; doi:10.1038/s41467-020-17459-z)
Supplement: Supplementary file 6 — Reporting Summary [file 41467_2020_17459_MOESM6_ESM.pdf]

nature research

Corresponding author(s): Francesco Maura, MD

Last updated by author(s): 2020/06/17

Reporting Summary

Nature Research wishes to improve the reproducibility of the work that we publish. This form provides structure for consistency and transparency in reporting. For further information on Nature Research policies, see Authors & Referees and the Editorial Policy Checklist.

Statistics

For all statistical analyses, confirm that the following items are present in the figure legend, table legend, main text, or Methods section.

n/a	Confirmed	
☐	☒	The exact sample size (*n*) for each experimental group/condition, given as a discrete number and unit of measurement
☐	☒	A statement on whether measurements were taken from distinct samples or whether the same sample was measured repeatedly
☐	☒	The statistical test(s) used AND whether they are one- or two-sided<br>*Only common tests should be described solely by name; describe more complex techniques in the Methods section.*
☐	☒	A description of all covariates tested
☐	☒	A description of any assumptions or corrections, such as tests of normality and adjustment for multiple comparisons
☐	☒	A full description of the statistical parameters including central tendency (e.g. means) or other basic estimates (e.g. regression coefficient) AND variation (e.g. standard deviation) or associated estimates of uncertainty (e.g. confidence intervals)
☐	☒	For null hypothesis testing, the test statistic (e.g. *F*, *t*, *r*) with confidence intervals, effect sizes, degrees of freedom and *P* value noted<br>*Give P values as exact values whenever suitable.*
☐	☒	For Bayesian analysis, information on the choice of priors and Markov chain Monte Carlo settings
☒	☐	For hierarchical and complex designs, identification of the appropriate level for tests and full reporting of outcomes
☐	☒	Estimates of effect sizes (e.g. Cohen's *d*, Pearson's *r*), indicating how they were calculated

Our web collection on statistics for biologists contains articles on many of the points above.

Software and code

Policy information about availability of computer code

Data collection	Publicly available published software were used for whole genome sequencing analysis as follows BWA-MEM v0.7.8, Caveman v1.13.2, Pindel v1.5.7, BRASS v4.012, Battenberg (https://github.com/Wedge-lab/battenberg)
Data analysis	Pylogenetic tree recontruction: hierarchical Dirichlet process (https://github.com/Wedge-Oxford/dpclust)<br>Mutational signatures were analyzed by SigProfiler (https://github.com/AlexandrovLab/SigProfilerExtractor) and mmsig (https://github.com/evenrus/mmsig)

For manuscripts utilizing custom algorithms or software that are central to the research but not yet described in published literature, software must be made available to editors/reviewers. We strongly encourage code deposition in a community repository (e.g. GitHub). See the Nature Research guidelines for submitting code & software for further information.

Data

Policy information about availability of data

All manuscripts must include a data availability statement. This statement should provide the following information, where applicable:

- Accession codes, unique identifiers, or web links for publicly available datasets
- A list of figures that have associated raw data
- A description of any restrictions on data availability

All data are available in the following public depositories: EGAS00001002111 and EGAS00001004404

Field-specific reporting

Please select the one below that is the best fit for your research. If you are not sure, read the appropriate sections before making your selection.

☒ Life sciences ☐ Behavioural & social sciences ☐ Ecological, evolutionary & environmental sciences

For a reference copy of the document with all sections, see nature.com/documents/nr-reporting-summary-flat.pdf

Life sciences study design

All studies must disclose on these points even when the disclosure is negative.

Sample size	In this study we included 4 multiple myeloma patients with multiple samples collected at autopsy (21 tumor sample). As validation we included 125 available whole exome sequencing from 51 patients. Sample size was defined according to the sequencing data availability. The WGS cohrt size was decided according to sample availability within the MSKCC Last Wish program.
Data exclusions	Four patients included in the whole exome sequencing cohor were removed because the DP failed due to either low CNA quality or the low sample purity.
Replication	All data can be entirely reproduced. All the sequencing data and bio-informatics codes are available in public depository
Randomization	No randomization was require for this study where we compared genomic data from samples collected before and after treatment. The study was not an experimental clinical treatment trial and hence no randomization was performed.
Blinding	No drugs were involved. Being a descriptive genomic study blinding was not applicable.

Reporting for specific materials, systems and methods

We require information from authors about some types of materials, experimental systems and methods used in many studies. Here, indicate whether each material, system or method listed is relevant to your study. If you are not sure if a list item applies to your research, read the appropriate section before selecting a response.

Materials & experimental systems

n/a	Involved in the study	
☒	☐	Antibodies
☒	☐	Eukaryotic cell lines
☒	☐	Palaeontology
☒	☐	Animals and other organisms
☐	☒	Human research participants
☒	☐	Clinical data

Methods

n/a	Involved in the study	
☒	☐	ChIP-seq
☒	☐	Flow cytometry
☒	☐	MRI-based neuroimaging

Human research participants

Policy information about studies involving human research participants

Population characteristics	The study involved the use of human samples, which were collected from patients enrolled in the "Last Wish Program" at MSKCC after written informed consent was obtained. Samples and data were obtained and managed in accordance with the Declaration of Helsinki.<br><br>Each tumor sample was collected from a different disease localization site and DNA was extracted from CD138+ purified cells. To avoid contamination related to late systemic disease dissemination, cells collected from skeletal muscles were used as matched normal controls. All normal samples were histologically reviewed to exclude microscopic foci of tumor. Tumor biopsies collected were commonly very cellular and only those with > 70% cellularity based on histologic review were selected for DNA extraction. To this cohort we added available and published whole exome sequencing data
Recruitment	Sample were collected from patients enrolled in the "Last Wish Program" at MSKCC. The Last Wish Program is not a clinical trial. It is an ongoing research biospecimen protocol (protocol #15-021, approved by the Institutional Review Board of MSKCC) which permits the postmortem collection of tissue and other samples from deceased patients, from whom consent for the protocol was obtained antemortem. The protocol allows for the performance of a broad range of research studies using the collected samples. We include all available samples without any selection bias.
Ethics oversight	All patients consented to autopsy and sample collection as a part of the "Last Wish Program" at Memorial Sloan Kettering Cancer Center (MSKCC) (protocol #15-021, approved by the Institutional Review Board of MSKCC)

Note that full information on the approval of the study protocol must also be provided in the manuscript.
